# Comparative analysis of *Acinetobacter baumannii* bloodstream isolates from children and adults in southern China

**DOI:** 10.3389/fmicb.2025.1684049

**Published:** 2025-10-14

**Authors:** Fen Xue, Hui Xu, Nan Gao, Cheng Li, Fan Fei, Rui Zhao, Tiantian Zhang, Shuaiyuan Liang, Xing Wang, Yanfeng Zhao, Xingran Du

**Affiliations:** ^1^Laboratory Medicine Center, The Second Affiliated Hospital of Nanjing Medical University, Nanjing, Jiangsu, China; ^2^Department of Pulmonary and Critical Care Medicine, The Second Affiliated Hospital of Nanjing Medical University, Nanjing, Jiangsu, China; ^3^Department of Pulmonary and Critical Care Medicine, The Affiliated Jiangning Hospital with Nanjing Medical University, Nanjing, Jiangsu, China; ^4^Department of Laboratory Medicine, Shanghai Children’s Medical Center, Shanghai Jiao Tong University School of Medicine, Shanghai, China

**Keywords:** *Acinetobacter baumannii*, antimicrobial resistance, virulence gene, whole-genome sequencing, blood infection

## Abstract

**Introduction:**

*Acinetobacter baumannii* (*A. baumannii*) is a major pathogen responsible for hospital-acquired bloodstream infections, with multidrug-resistant (MDR) strains posing severe therapeutic challenges. Neonates are particularly vulnerable, with infections often associated with high morbidity and mortality. Thisstudy aimed to compare the genomic and phenotypic characteristics of A. baumannii isolates from children and adults.

**Methods:**

A total of 77 blood isolates of *A. baumannii* were collected, including 42 from children and 35 from adults. Antimicrobial susceptibility testing against 14 agents was performed. Whole-genome sequencing (WGS) was used for multilocus sequence typing (MLST), antimicrobial resistance gene and virulence gene detection, and phylogenetic analysis based on core-genome single-nucleotide polymorphisms (SNPs). Key resistance mechanisms (β-lactamase production and multidrug efflux pumps) and virulence factors (porins, lipopolysaccharides, metal acquisition systems, and secretion systems) were examined.

**Results:**

Carbapenem resistance was significantly higher in pediatric isolates (97.6%) compared with adult isolates (65.7%). Adult isolates exhibited greater diversity in OXA-type carbapenemases. Virulence gene analysis revealed widespread distribution of porins, lipopolysaccharide synthesis genes, metal acquisition systems, and type VI secretion system components in both groups, with a higher detection rate in pediatric isolates. The majority of isolates belonged to ST2 (89.6%) and carried the blaOXA-23 gene, while ST466 and ST57 were exclusively identified in adult isolates.

**Conclusion:**

These findings demonstrate age-related differences in the resistance and virulence profiles of *A. baumannii* bloodstream isolates. Pediatric isolates exhibited higher carbapenem resistance and virulence gene prevalence, whereas adult isolates showed greater clonal diversity. This comparative analysis enhances understanding of *A. baumannii* pathogenesis across age groups and provides insights for guiding empirical therapy and strengthening infection control strategies.

## Introduction

*Acinetobacter baumannii (A. baumannii)*, a Gram-negative opportunistic pathogen, is a major cause of hospital-acquired infections, including pneumonia, bloodstream infections (BSIs), urinary tract infections, and meningitis (Papazachariou et al., 2024). Notably, the carbapenem resistance rate of *A. baumannii* in China has shown a consistent upward trend in recent years, with infections caused by carbapenem-resistant *A. baumannii* (CRAB) being associated with persistently high mortality rates (Xu et al., 2018). This organism has emerged as a critical global threat due to its role in multidrug-resistant (MDR) nosocomial infections (Cherubini et al., 2022). Among Gram-negative bacterial infections, BSIs represent one of the most severe clinical presentations, with morbidity and mortality rates further exacerbated by the growing prevalence of MDR pathogens (Aydın Teke et al., 2017; Diekema et al., 2019). Surveillance data from U. S. intensive care units (ICUs) indicate that *A. baumannii* BSIs carry a crude mortality rate of 34%, ranking third among bacterial BSIs in lethality (Wisplinghoff et al., 2000). Although community-acquired bloodstream infections caused by *A. baumannii* remain relatively rare, outbreaks among immunocompromised populations have been documented, often leading to poor clinical outcomes (Chen et al., 2018). Of particular concern is the heightened vulnerability of infants admitted to the neonatal intensive care unit (NICUs), who face prolonged and extensive exposure to antibiotics and the hospital environments, resulting in a 30% mortality rate associated with MDR Gram-negative bacterial (MDR-GNB) colonization and infection (Folgori et al., 2018; Stapleton et al., 2016; Hartz et al., 2015). This underscores the necessity for targeted infection prevention measures in this high-risk population. Current research on *A. baumannii* predominantly focuses on antimicrobial resistance patterns or hospital transmission dynamics within single patient group, leaving underexplored the comparison between adult and pediatric BSI isolates.

Specifically, systematic investigations into resistance gene profiles, virulence factor distributions, and strain phylogeny remain scarce, directly impeding the development of precision treatment protocols and optimized infection control measures. To address this, we analyzed clinical data and whole-genome sequencing results from 77 BSI isolates collected at our institution. By employing molecular epidemiological approaches, this study systematically compares antimicrobial resistance traits, virulence gene profiles, and clonal relatedness between adult and pediatric *A. baumannii* strains, aiming to generate evidence-based guidance for clinical antimicrobial therapy and hospital infection control.

## Materials and methods

### Strain selection

Clinical data and whole blood samples from patients with *A. baumannii* BSIs were collected prospectively from the Departments of Laboratory Medicine at Shanghai Children’s Medical Center and the Second Affiliated Hospital of Nanjing Medical University between 2018 and 2024. The cohort comprised 42 pediatric patients (aged from 1 month to 13 years) and 35 adult patients (predominantly elderly individual ssuffering from complications such as kidney disease, diabetes, hypertension, or heart disease). All isolates were transferred to the clinical microbiology laboratory and analyzed according to the Clinical and Laboratory Standards Institute (CLSI) guidelines. Only one *A. baumannii* strain per patient was included in the study.

### Strain identification

All isolates were collected from Nanjing Medical University Second Affiliated Hospital and Shanghai Children’s Center between 2016 and 2024. Positive blood culture samples were streaked on blood and chocolate agar plates (Comag, Shanghai, China) and incubated at 37 °C in 5% CO₂ for 18–24 h. Distinct colonies were examined for morphology, hemolysis, and growth on chocolate agar, and confirmed as Gram-negative bacilli by Gram staining. Species identification was performed using matrix-assisted laser desorption/ionization time-of-flight mass spectrometry (MALDI-TOF MS; Smart MS database), following the manufacturer’s guidelines. Only isolates with identification scores ≥2.0 were included for antimicrobial susceptibility testing and molecular analysis. *Escherichia coli* ATCC 8739 served as the quality control strain.

### Antimicrobial susceptibility testing

All isolates underwent antibiotic susceptibility testing using the Vitek-2 Compact system with the VITEK 2 AST-N335 susceptibility card (Menier, France). The minimum inhibitory concentrations (MICs) of 15 antibiotics were determined via the microbroth dilution method, following CLSI-M100 guidelines (Ii et al., 2023). Tested antibiotics included: Piperacillin/Tazobactam (TZP), Ceftazidime (CAZ), Cefoperazone/Sulbactam (CAS), Cefepime (FEP), Imipenem (IPM), Meropenem (MEM), Amikacin (AMK), Tobramycin (TOB), Gentamicin (GEN), Ciprofloxacin (CIP), Levofloxacin (LEV), Tigecycline (TGC), Polymyxin B (PB), Sulfamethoxazole/Trimethoprim (SXT) Isolates were stored at −80 °C for further analysis.

### Bacterial whole-genome sequencing

Whole-genome sequencing was performed on all 77 isolates. Using high-quality DNA samplesto construct fragment libraries. Sequencing was completed by Shanghai Biozeron Biotechnology Co., Ltd. (Shanghai, China). According to the standard genomic DNA library preparation protocol, paired-end libraries with insert fragment sizes of approximately 400 bp were prepared. After the library passes quality control, the different libraries are pooled according to the requirements of effective concentration and target sequencing data volume, then subjected to next-generation sequencing. The basic principle of sequencing is sequencing by synthesis. Four types of fluorescently labeled dNTPs, DNA polymerase, and adapter primers are added in the sequencing flow cell for amplification. The sequencer captures the fluorescence signal and uses computer software to convert the light signal into sequencing peaks, thereby obtaining the sequence information of the fragment to be tested.

### Multilocus sequence typing

The FASTQ format generated from the whole-genome sequencing results of 77 *A. baumannii* isolates was used for subsequent analysis. For sequence type (ST) determination, the filtered FASTQ files of each isolate were uploaded to the PubMLST online platform^1^ and analyzed using the *A. baumannii* Pasteur multilocus sequence typing (MLST) scheme. This scheme characterizes isolates by identifying the allelic profiles of seven housekeeping genes: gltA, gyrB, gdhB, recA, cpn60, gpi, and rpoD.

### Core genome SNP phylogenetic tree

Using Parsnp (v1.2) to construct the core genome single-nucleotide polymorphism (SNP) phylogenetic tree of the strains. Among them, strains A-ab24, A-ab2530, A-ab255, A-ab3322, A-ab3592, A-ab4388, and C-ab34 are genetically distant from the other 70 strains and were not included in the construction of the core genome SNP phylogenetic tree. The tree was visualized and annotated using the Interactive Tree of Life (iTOL) platform.

### Drug resistance gene

Antimicrobial resistance genes (ARGs) were annotated using the Comprehensive Antibiotic Resistance Database (CARD) and Resistance Gene Identifier (RGI). CARD provides annotations and functional information about antibiotic resistance genes, while RGI is primarily used to detect and predict genes related to antibiotic resistance in bacterial genomes or transcriptomes.

### Virulence gene profiling

Virulence factors were identified via the software DIAMOND BLASTp (*E*-value: 1 × 10^−5^; Identity: ≥70%; Coverage: ≥90%), the virulence factors of the strain’s genome were identified and analyzed through the Virulence Factor Database (VFDB)^2^ (Hong et al., 2023).

### Statistical analysis

All statistical analyses were conducted using IBM SPSS Statistics 27 (IBM Corp, United States). Categorical variables are presented as counts (percentages), and continuous variables as mean ± standard deviation (SD). The comparison of categorical variables between groups was conducted using the chi-square test or Fisher’s exact tests (categorical variables) or Mann–Whitney U tests (non-normally distributed continuous variables). Odds ratios (ORs) with 95% confidence intervals (CI) were calculated. Two-tailed *p* values < 0.05were considered statistically significant.

## Results

### Univariate analysis comparing pediatric and adult patients

A classification and univariate analysis was performed on 77 *A. baumannii* strains identified from blood samples, including 35 from adults and 42 from pediatric patients. Among the pediatric isolates, 37 (88.1%) originated from the Pediatric Intensive Care Unit (PICU), while the remaining 4 were from the cardiothoracic and oncology departments (Table 1). Previous studies have reported that primary diseases, immunodeficiencies, broad-spectrum antibiotics use, and invasive procedures increase the incidence of bloodstream infections in children (Hong et al., 2023). In this study, 15 children (35.7%) exhibited pneumonia symptoms, 7 children (16.7%) were diagnosed with leukemia, and 7 children (16.7%) had congenital heart disease. Additionally, studies have shown that prematurity is an independent risk factor for carbapenem-resistant *A. baumannii* bacteremia (Punpanich et al., 2012). Notably, 25 isolates (59.5%) were from infants under 1 year old. In contrast, most adult patients were elderly, with an average age of 70 ± 13 years. Fewer patients were from the ICU (31.4%), and pneumonia was the main manifestation (17.1%).

**Table 1:** Univariate analysis was used to compare the differences of bloodstream infection between the children and adults.

Variables	Children	Adult	Univariate analysis	
			OR (95% CI)	*P* value
Age, mean ± SD (years)	2.8 ± 4.0	70.2 ± 13.7		**<0.001**
Male sex (*n*, %)	28 (66.7)	26 (74.3)	0.69 (0.26–1.87)	0.467
Distribution of departments (*n*, %)				
ICU	37 (88.1)	11 (31.4)	16.15 (4.98–52.30)	**<0.001**
Department of Hematology	3 (7.14)	7 (20.0)	0.31 (0.07–1.30)	0.183
Thoracic Surgery	2 (4.8)	0	1.88 (1.52–2.31)	0.498
Respiratory Medicine Department	0	3 (8.6)	2.31 (1.78–3.00)	0.089
Other Departments	0	14 (40.0)	3.00 (2.12–4.25)	**<0.001**
Specimen source (*n*, %)				
Blood	42 (100)	35 (100)	1.20 (0.02–61.89)	1
Comorbidities (*n*, %)				
Pneumonia	15 (35.7)	6 (17.1)	2.69 (0.91–7.92)	0.068
Leukemia	7 (16.7)	1 (2.9)	6.80 (0.79–58.25)	0.065
CHD	7 (16.7)	0	15.0 (0.83–273.55)	1

ICU, Intensive Care Unit; CHD, Congenital Heart Disease; OR, Odds Ratio; CI, Confidence Interval. Bold values indicate statistically significant differences (*p* < 0.001).

### Antimicrobial resistance profile

Antimicrobial susceptibility testing of 77 isolates of *A. baumannii* revealed high rates of resistance to imipenem and meropenem (64, 83.1%) and piperacillin/tazobactam and ciprofloxacin (62, 80.5%), exceeding 80% (Table 2). Resistance analysis indicated that *A. baumannii* isolates exhibited strong resistance to β-lactam antibiotics, carbapenems, and third-generation cephalosporins. In the pediatric group, resistance rates for ciprofloxacin, piperacillin/tazobactam, carbapenems, and third-generation cephalosporins all exceeded 90%. The resistance rates to imipenem, meropenem, and ceftazidime reached as high as 97.6% (41 strains). In this study, the overall resistance rate of *A. baumannii* to imipenem and meropenem was 83.1%. Further analysis revealed that the resistance rate to both antibiotics was significantly higher in the pediatric group (97.6%) than in the adult group (65.7%) (*p* < 0.001). These findings indicate a more severe carbapenem resistance profile in pediatric *A. baumannii* infections. Therefore, it can be seen that pediatric isolates demonstrated higher resistance overall, posing greater risks of mortality and complications, underscoring the urgency of infection control measures.

**Table 2:** Antimicrobial susceptibility of *Acinetobacter baumannii* lsolates.

Antimicrobial	Resistance, *n* (%)			*P* value		
	Total (*n* = 77)	Children (*n* = 42)	Adults (*n* = 35)	*P* (C vs A)	*P* (C vs T)	*P* (A vs T)
Piperacillin/tazobactam	62 (80.5)	39 (92.9)	23 (65.7)	0.003	0.073	0.090
Ceftazidime	53 (68.8)	41 (97.6)	12 (34.3)	<0.001	<0.001	<0.001
Cefoperazone/sulbactam	37 (48.1)	29 (69.0)	8 (22.9)	<0.001	0.028	0.012
Cefepime	63 (81.8)	40 (95.2)	23 (65.7)	<0.001	0.040	0.061
Imipenem	64 (83.1)	41 (97.6)	23 (65.7)	<0.001	0.019	0.040
Meropenem	64 (83.1)	41 (97.6)	23 (65.7)	<0.001	0.019	0.040
Amikacin	9 (11.7)	8 (19.0)	1 (2.9)	0.065	0.273	0.245
Tobramycin	47 (61.0)	32 (76.2)	15 (42.9)	0.003	0.095	0.073
Gentamicin	49 (63.6)	35 (83.3)	14 (40.0)	<0.001	0.024	0.019
Ciprofloxacin	62 (80.5)	39 (92.9)	23 (65.7)	0.003	0.073	0.090
Levofloxacin	38 (49.4)	29 (69.0)	9 (25.7)	<0.001	0.038	0.019
Tigecycline	1 (1.3)	0	1 (2.9)	0.455	1	0.529
Polymyxin B	1 (1.3)	1 (2.4)	0	1	1	1
Sulfamethoxazole/trimethoprim	46 (59.7)	37 (88.1)	9 (25.7)	<0.001	0.001	<0.001

C vs A, Comparison between adults and children; C vs T, Children compared with the total; A vs T, Adults compared with the total.

### Distribution of antibiotic resistance genes

The resistance gene profiles of *A. baumannii* isolates from adults and children were shown in Tables 3, 4. In terms of carbapenem resistance genes, differences were observed between pediatric and adult isolates. In the pediatric group, the prevalence of OXA-23 (41/42, 97.6%), OXA-66 (39/42, 92.9%), and OXA-167 (40/42, 95.2%) were exceptionally high. These three genes, are key contributors to the markedly high imipenem resistance rate (97.6%) in pediatric isolates. Of particular note is that NDM-1, OXA-58, and OXA-510 were detected simultaneously in the pediatric isolate C-ab14, but OXA-23 was not detected. Multilocus sequence typing (MLST) results suggest that this strain is a novel ST (ST-). In contrast, adult isolates, while still carrying a significant proportion of OXA-23 (21/35, 60%), OXA-66 (24/35, 68.6%) and OXA-167 (25/35, 71.4%), exhibited a lower overall prevalence and a wider diversity of OXA gene types. These included sporadic detections of novel carbapenemases such as OXA-259, OXA-421, OXA-500, and OXA-532, suggesting the emergence of new resistance determinants within the adult population (Table 3). The high resistance rate of ciprofloxacin in the drug susceptibility test is consistent (Table 2). This resistance mechanism is related to the antibiotic efflux pump (Jacoby et al., 2014). The genes monitored through sequencing mainly belong to the resistance-nodulation-cell division (RND) antibiotic efflux pump, such as adeL/G, while the *abaF* (42/42, 100%) observed in children is a major facilitator superfamily (MFS) efflux pump, which is the reason for the resistance to phosphonic acid antibiotics.

**Table 3:** Prevalence of acquired *bla*_OXA_ genes in children and adult *A. baumannii* isolates.

*bla*_OXA_-like genes detected	Children (*n*, %)	Adults (*n*, %)
OXA-23	41 (97.6)	21 (60.0)
OXA-64	0	1 (2.9)
OXA-66	39 (92.9)	24 (68.6)
OXA-69	1 (2.4)	0
OXA-96	1 (2.4)	0
OXA-106	0	1 (2.9)
OXA-120	1 (2.4)	0
OXA-167	40 (95.2)	25 (71.4)
OXA-259	0	1 (2.9)
OXA-409	0	1 (2.9)
OXA-421	0	2 (5.7)
OXA-500	0	1 (2.9)
OXA-502	0	1 (2.9)
OXA-510	1 (2.4)	0
OXA-532	0	1 (2.9)

**Table 4:** Frequencies of virulence genes and drug resistance genes among *A. baumannii* isolates in blood samples obtained from adults and children.

Gene	*A. baumannii* (*n*, %)	Children (*n*, %)	Adults (*n*, %)	*p*-value
	(*n* = 77)	(*n* = 42)	(*n* = 35)	
Virulence genes				
pilA	62 (80.5)	40 (51.9)	22 (28.6)	**<0.001**
fimV	72 (93.5)	42 (54.5)	30 (39.0)	**0.016**
gspO/pilD	76 (98.7)	42 (54.5)	34 (44.2)	0.455
csuA/B	66 (85.7)	38 (49.4)	28 (36.4)	0.191
rmlB	6 (7.8)	3 (3.9)	3 (3.9)	1
Drug resistance genes				
AAC(6′)-Ib′	24 (31.2)	19 (24.7)	5 (6.5)	**0.004**
Adec	65 (84.4)	40 (51.9)	25 (32.5)	**0.004**
ADC-73	27 (35.1)	13 (16.9)	14 (18.2)	0.407
APH(3″)-Ib	23 (29.9)	16 (20.8)	7 (9.1)	0.084
catB8	27 (35.1)	20 (26.0)	7 (9.1)	**0.011**
Smef	73 (94.8)	42 (54.5)	31 (40.3)	**0.039**
tet(B)	61 (79.2)	39 (50.6)	22 (28.6)	**0.001**
adeG	75 (97.4)	41 (53.2)	34 (44.2)	1
AAC(6′)-Ib′	24 (31.2)	19 (24.7)	5 (6.5)	**0.004**
ADC-30	36 (46.8)	26 (33.8)	10 (13.0)	**0.004**
armA	52 (67.5)	31 (40.3)	21 (27.3)	0.198
adeC	65 (84.4)	40 (51.9)	25 (32.5)	**0.004**
arlR	12 (15.6)	2 (2.6)	10 (13.0)	**0.004**
catB8	27 (35.1)	20 (26.0)	7 (9.1)	**0.011**
msrE	55 (71.4)	33 (42.9)	22 (28.6)	0.129
MphE	55 (71.4)	33 (42.9)	22 (28.6)	0.129
cprR	11 (14.3)	1 (1.3)	10 (13.0)	**0.001**
mexN	73 (94.8)	41 (53.2)	32 (41.6)	0.482
smeF	73 (94.8)	42 (54.5)	31 (40.3)	**0.039**
acrD	74 (96.1)	42 (54.5)	32 (41.6)	0.089
sul1	27 (35.1)	20 (26.0)	7 (9.1)	**0.011**
adeL/G	76 (98.7)	42 (54.5)	34 (44.2)	0.455
AbaF	77 (100.0)	42 (54.5)	35 (45.5)	1

Bold values indicate statistically significant differences (*p* < 0.001).

Following this, macrolide resistance genes were also commonly detected, such as *macA* (77/77, 100%), *mphE*, and *msrE* (55/70, 71.4%), which were prevalent (Table 4). *MphE* and *msrE* belong to the msr-type ABC-F protein and macrolide phosphotransferase gene families, respectively, and they mediate different resistance mechanisms.

For aminoglycoside antibiotics such as gentamicin, tobramycin, and amikacin, the distribution among adults and children is highest in the chart, especially the high resistance rates in children for gentamicin (35/42, 83.3%) and tobramycin (32/42, 76.2%) (Table 2). These drugs are contraindicated in children under six due to toxicity, and the observed high resistance rates further limit their clinical applicability in pediatric cases, necessitating alternative therapeutic strategies.

### Homology and ST typing

**Figure 1:**
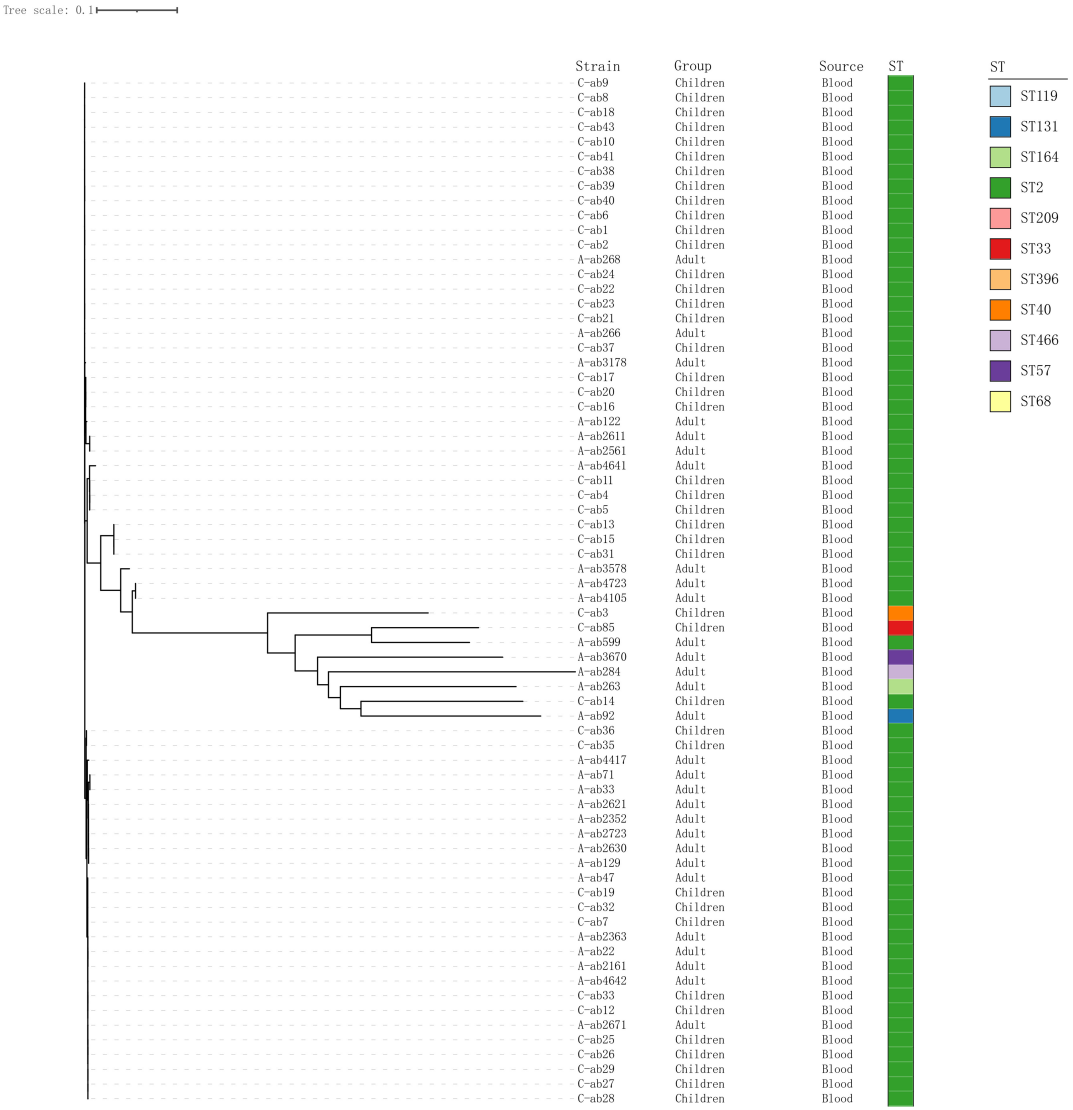
Phylogenetic tree of 70 isolates. Construct a phylogenetic tree based on whole-genome SNPs using the maximum likelihood method, and display the source of strains and ST typing on the tree.

The phylogenetic tree shows the evolutionary relationships among the sample strains (Figure 1). Overall, the strains (C-ab3, C-ab85, A-ab599, A-ab3670, A-ab284, A-ab263, C-ab14, A-ab92) are relatively close in position on the tree, indicating that they are more closely related phylogenetically, with smaller genomic differences. Through MLST typing, a total of 11 ST types were detected (ST119, ST131, ST164, ST2, ST209, ST33, ST396, ST40, ST466, ST57, ST68), as well as 2 unidentified types (ST-). Among them, ST131, ST209, ST33, ST396, ST466, and ST57 have not been reported in the literature; ST2 (62/77, 80.5%) accounted for the highest proportion in all samples and carry *bla*_OXA-23_. We isolated an ST164 strain from blood, which carried *bla*_OXA-23_ and *bla*_OXA-91_, and the plasmid carried tet (39). Additionally, ST33 and ST40 types were relatively concentrated on the phylogenetic tree, forming independent evolutionary branches. On the other hand, the ST-type strains could not match any known types in the existing database, possibly representing new ST types or those not yet included in the database, requiring further research to determine their genetic background.

### Virulence gene analysis

**Figure 2:**
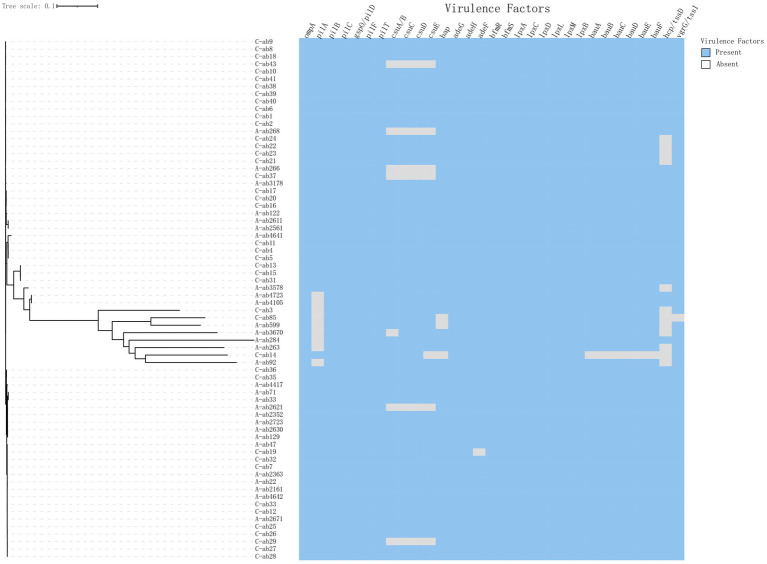
Heatmap of bacterial virulence factors in adult and children groups.

Conduct virulence gene profiling analysis of *A. baumannii* isolates from children and adults to evaluate genes associated with key virulence factors such as fimbriae, biofilm formation, lipopolysaccharide synthesis, iron acquisition, and type III and type VI secretion systems (Figure 2). All strains contain the Outer Membrane Protein A (*ompA*) gene (77/77, 100%), which is an important virulence factor mediating antibiotic resistance, biofilm formation, and host interactions (Mortensen and Skaar, 2012; Scribano et al., 2024). The gene deletion portions shown in the figure mostly come from the Csu pili (*csuA*/*B* (66/77, 85.7%), *csuC*, *csuD*, *csuE*) and *hcp*/*tssD* (60/77, 77.9%) genes (Table 4). Analysis of the T6SS components hcp/tssD showed that these genes are dispersed, with 7 strains (16.7%) in children and 5 strains (14.2%) in adults missing them. In addition, this study detected the presence of the *rmlB* gene in three pediatric and three adult isolates. Vertically, the *pilA* (62/77, 80.5%) deletions were concentrated in a few larger branches of bacteria (Figure 2), which are fimbrial-related genes. The expression of the pilA gene aids in the adhesion and colonization of *A. baumannii* during the early stages of infection, thereby enhancing its pathogenicity (Cerqueira et al., 2014).

## Discussion

Population-based BSI surveillance projects from various regions indicate that *Escherichia coli* and *Staphylococcus aureus* are the two most common BSI pathogens, with estimated incidence rates of 35/100,000 and 25/100,000, respectively (Diekema et al., 2019). However, in the past 5 years, the clinical significance of *A. baumannii* as a nosocomial pathogen has been increasing, with isolation rates as high as 20–40% in hospital-acquired BSI cases (Yang et al., 2019; Wareth et al., 2020). Patients with *A. baumannii* BSI are more often hospitalized in ICU and more frequently receive mechanical ventilation. In this study, compared to adults, 88.1% of Abn-BSI pediatric came from the PICU. Among the 45 Abn-BSI pediatric, 35.7% (15/45) had pneumonia, and 16.7% (7/45) had acute leukemia and congenital heart disease. The underdevelopment of the immune system and immature immune defense functions in neonates are significant factors leading to bloodstream infections, resulting in a higher incidence rate (Hong et al., 2023). 31.4% (11/35) of the adults came from the ICU, with an mean age of approximately 70 years. The clinical diagnoses were mostly severe pneumonia, malignant tumors, or post-brain surgery, indicating a higher prevalence of underlying diseases.

The rapid development of antibiotic resistance and/or multidrug resistance patterns has attracted global attention (Nogbou et al., 2022), with significant differences in the sensitivity to antimicrobial agents and the infection rates in epidemics (Table 1). Our data show that the resistance rate to carbapenem antibiotics is as high as 83.1% for all strains, which is consistent with the results of earlier studies (Nogbou et al., 2021). The resistance rates for adults and children are 65.7 and 97.6%, respectively. Several factors may explain this discrepancy: First, identifying pathogens early in critically ill children is challenging, making them more likely to receive broad-spectrum antibiotics such as carbapenems and broad-spectrum cephalosporins as empirical therapy. Second, children in PICUs—particularly neonates and infants—possess immature immune systems and frequently require antibiotics alongside invasive devices like central venous catheters and ventilators. This environment facilitates bacterial adaptation into low-metabolism persister populations, which exhibit heightened resistance to carbapenems and cephalosporins. Resistance gene clusters are enriched in pediatric isolates. For instance, this study found pediatric patients frequently carried the blaOXA-23 carbapenemase gene (97.6%) and OXA-167 (OXA-23-like, 95.2%). Previous studies have demonstrated that OXA-23-like enzymes are the most prevalent acquired carbapenemases in *A. baumannii*, conferring high-level resistance to carbapenems (Evans and Amyes, 2014), while OXA-51-like, though intrinsic, can significantly enhance resistance when combined with insertion sequences such as ISAba1 (Chan et al., 2022; Brown et al., 2005). It is worth noting that although the imipenem resistance rate of adult isolates is lower than that of children, adult isolates exhibit a more diverse spectrum of OXA-type carbapenemases compared to pediatric isolates such as OXA-259, OXA-409, OXA-421, OXA-500, OXA-502, and OXA-532. This may stem from heterogeneity in the genetic background of adult-derived *A. baumannii* clones within AbaR/AbGRI-type resistance islands and transposable elements like ISAba1/Tn2006, which carry different *bla_OXA_* genes. This heterogeneity amplifies the diversity of detectable OXA-type carbapenemases within the same healthcare facility (Douraghi et al., 2020). Concurrently, studies indicate that high resistance rates in pediatric ICUs are primarily driven by the expansion of a single clone, such as GC2, carrying *bla_OXA-23_* and enhanced by upstream expression via ISAba1. While this increases overall carbapenem resistance rates, it reduces OXA-type diversity due to clonal homogeneity (Yousefi Nojookambari et al., 2021). In addition, we identified a novel sequence type (ST-) pediatric isolate (C-ab14) harboring NDM-1, OXA-58, and OXA-510 simultaneously, but notably lacking OXA-23. Although previous reports have documented the coexistence of NDM-1 and OXA-58 in individual cases (Rodrigues et al., 2024), the combination of these three carbapenemase genes within a novel ST has not been previously described.

The resistance rates of *A. baumannii* to fluoroquinolone antibiotics vary significantly. Wisplinghoff et al. (2012) reported a levofloxacin resistance rate of 33.2%, while *A. baumannii* from a tertiary hospital in Pretoria, South Africa, showed an 83% resistance rate to ciprofloxacin (Nogbou et al., 2021), indicating high resistance. In contrast, *A. baumannii* isolated from patients with nosocomial pneumonia or ventilator-associated pneumonia in a hospital in Vietnam (Wareth et al., 2021) exhibited resistance rates exceeding 90% to cephalosporins, fluoroquinolones, and carbapenems. In comparison, our study shows both similarities and differences in resistance patterns. The overall resistance rates to levofloxacin and ciprofloxacin were 49.4 and 80.5%, respectively. However, subgroup analysis revealed that in pediatric patients, the resistance rates to levofloxacin and ciprofloxacin were 69 and 92.9%, respectively, while in adult patients, the corresponding rates were 25.7 and 65.7%.

It has been determined that tigecycline is suitable for the treatment of bloodstream infections owing to Gram-negative bacteria (GNB) (Jean et al., 2022). In our study results, most isolates retained susceptibility to tigecycline. Polymyxins, although effective, serve as second-line therapies due to nephrotoxicity and neurotoxicity risks (Nang et al., 2021). The currently preferred backbone for the treatment of carbapenem-resistant colistin-resistant *A. baumannii* is sulbactam. However, the optimal combination regimen remains unclear. New agents like sulbactam-durlobactam, a bactericidal β-lactamase inhibitor combination, have demonstrated promising activity against CRAB, yet their high cost and limited availability currently restrict routine use (Kaye et al., 2023). This highlights the urgent need for rational antibiotic stewardship.

Virulence factors such as fimbriae associated with biofilm formation and adhesion are very common in the isolated strains, indicating their role in bloodstream infections.

According to MLST typing analysis of *A. baumannii*, we revealed that ST2 was the predominant clade, accounting for 80.5% (62/77) of the isolates, all of which carried the carbapenemase gene blaOXA-23. This clade dominates in bloodstream infections, highlighting its significance for clinical diagnosis and treatment, and demonstrates strong adaptability and virulence across different age groups. Sabrina et al. (Cherubini et al., 2022) also isolated ST2-type *A. baumannii* from patients with bacterial sepsis. Notably, ST2 belongs to the International Clone (IC2), one of the most common clonal types in global *A. baumannii* genome sequencing. Additionally, this study also isolated a strain of ST164, which also carries the bla-OXA23 carbapenemase gene. This is a newly emerging carbapenem-resistant *A. baumannii* that has attracted global attention (Liu et al., 2024).

The csuA/BCDE operon is essential for fimbriae synthesis in *A. baumannii*. Fimbriae are crucial components of mature biofilms, mediating bacterial adhesion and promoting disease development, and play a significant role in attachment to abiotic surfaces and biofilm formation (Ahmad et al., 2023; Ramezanalizadeh et al., 2020). We found that 87% (67/77) of the strains in the isolates contained the cus operon, highlighting the importance of biofilm-related genes in enhancing the pathogenicity of *A. baumannii*, which is also one of the factors contributing to its high virulence. The study also found differences in the prevalence of certain virulence genes between adults and children. For example, *pilA* (40/42, 95.2%) was detected more frequently in pediatric isolates, posing significant challenges to current CRAB treatment regimens for children (Lee et al., 2017). In addition, the rmlB gene was detected in a subset of adult and pediatric isolates. This gene, involved in lipopolysaccharide biosynthesis, plays a critical role in maintaining outer membrane integrity and immune evasion mechanisms (Li et al., 2022). Its functional role in *A. baumannii* bloodstream infections warrants further investigation, especially given its association with LPS-related immune modulation.

This study primarily analyzed resistance genes and virulence factors at the genomic level, without further exploring their functional mechanisms. It also faced limitations such as insufficient sample size. Future research should also incorporate larger datasets and evaluate the therapeutic potential of novel agents such as sulbactam-durlobactam in pediatric CRAB infections.

## Conclusion

Overall, this study’s comparative analysis of antibiotic resistance, genetic, and virulence profiles of 77 strains of *A. baumannii* isolated from adult and pediatric blood samples revealed that the Carbapenem resistance rate in pediatric isolates (97.6%) was significantly higher than in adults (65.7%). Virulence and resistance genes were detected at higher rates in children, while oxa-type carbapenemases exhibited greater diversity in adults. This finding contributes to a deeper understanding of the pathogenicity of *A. baumannii*. Concurrently, the presence of multidrug resistance and virulence factors underscores the need for new treatment strategies and ongoing surveillance to address *A. baumannii* infections in clinical settings.

## Importance

*A. baumannii* is one of the most important pathogens of hospital-acquired infections, which can cause a variety of clinical diseases, among which bloodstream infections are more serious and are characterized by difficulties in treatment and high mortality. In this study, comparative analyses of antibiotic resistance, virulence factors, and resistance genes were performed on *A. baumannii* blood isolates from children and adult patients. The results showed that child isolates had higher rates of antimicrobial resistance, virulence levels, and resistance gene detection than adult isolates; whereas adult isolates exhibited more diverse OXA-type carbapenemases. These findings provide new insights into the differences in disease severity and prognosis following *A. baumannii* infection in different age groups, and highlight the importance of targeted surveillance and optimisation of antimicrobial use in patients with bloodstream infections.

## Data Availability

The raw sequencing data have been uploaded to the NCBI SRA database, and the accession number assigned is SRP616910.

## Footnotes

^1^ https://pubmlst.org/

^2^ http://www.mgc.ac.cn/VFs/
